# Achieving Radiation Tolerance through Non-Equilibrium Grain Boundary Structures

**DOI:** 10.1038/s41598-017-12407-2

**Published:** 2017-09-25

**Authors:** Gregory A. Vetterick, Jacob Gruber, Pranav K. Suri, Jon K. Baldwin, Marquis A. Kirk, Pete Baldo, Yong Q. Wang, Amit Misra, Garritt J. Tucker, Mitra L. Taheri

**Affiliations:** 1 Department of Materials Science and Engineering, Drexel University, Philadelphia, PA 19104 USA; 2 Center for Integrated Nanotechnologies, Los Alamos National Laboratory, Los Alamos, NM 87545 USA; 3 IVEM-Tandem Facility, Argonne National Laboratory, Argonne, IL 60439 USA; 4 Ion Beam Materials Laboratory, Materials Science and Technology Division, Los Alamos National Laboratory, Los Alamos, NM 87545 USA; 5 TerraPower, LLC, Bellevue, WA 98005 USA; 6 Department of Materials Science and Engineering, University of Michigan, Ann Arbor, Michigan, 48109 USA; 7 Present Address: Department of Mechanical Engineering, Colorado School of Mines, Golden, CO 80401 USA

## Abstract

Many methods used to produce nanocrystalline (NC) materials leave behind non-equilibrium grain boundaries (GBs) containing excess free volume and higher energy than their equilibrium counterparts with identical 5 degrees of freedom. Since non-equilibrium GBs have increased amounts of both strain and free volume, these boundaries may act as more efficient sinks for the excess interstitials and vacancies produced in a material under irradiation as compared to equilibrium GBs. The relative sink strengths of equilibrium and non-equilibrium GBs were explored by comparing the behavior of annealed (equilibrium) and as-deposited (non-equilibrium) NC iron films on irradiation. These results were coupled with atomistic simulations to better reveal the underlying processes occurring on timescales too short to capture using *in situ* TEM. After irradiation, NC iron with non-equilibrium GBs contains both a smaller number density of defect clusters and a smaller average defect cluster size. Simulations showed that excess free volume contribute to a decreased survival rate of point defects in cascades occurring adjacent to the GB and that these boundaries undergo less dramatic changes in structure upon irradiation. These results suggest that non-equilibrium GBs act as more efficient sinks for defects and could be utilized to create more radiation tolerant materials in future.

## Introduction

Nanocrystalline materials possess a high volume fraction of grain boundaries that can act as defect sinks to reduce the amount of damage retained after irradiation. This effect has already been shown to reduce resistivity changes in irradiated Au^1,2^, to reduce amorphization of MgGa_2_O_4_^3^ and TiNi^4^, and has been shown by TEM to reduce defect clustering in ZrO_2_^5^, Pd^5^, Cu–0.5A_2_O_3_^6^, and Ni^6,7^. The precise structure of grain boundaries in nanoscale materials is still of considerable debate, and may vary greatly depending on the process used to produce the material. For example, nanocrystalline materials produced by severe plastic deformation (SPD) contain large fractions of non-equilibrium grain boundaries, as do thin films produced by physical vapor deposition methods, such as sputtering, which are highly non-equilibrium processes. These boundaries contain excess free volume and higher energy than their equilibrium counterparts. Excess free volume in grain boundaries has been shown to have a clear effect on defect absorption and annihilation^8,9^. Since non-equilibrium grain boundaries have increased amounts of both strain and free volume, these boundaries may act as a more efficient sink than their equilibrium counterparts with identical 5 degrees of freedom^10^. Non-equilibrium boundaries may therefore provide a more effective sink for the excess interstitials and vacancies produced in a material under irradiation. SPD is commonly used to produce ultrafine grain and nanocrystalline ferritic-martensitic steels for advanced nuclear reactor applications; therefore, many of these materials contain large numbers of non-equilibrium grain boundaries. Thus, quantifying the role of grain boundary equilibrium in its ability to act as a defect sink is crucial to predicting the effectiveness of a nanocrystalline material as a radiation tolerant material.

Grain boundaries in polycrystalline materials were originally theorized to be amorphous (e.g., Ruder^11^ and McLean^12^). Today, it has been firmly established that the structure of grain boundaries in microcrystalline materials is crystal-like^13–16^ and defined by a series of dislocations or structural units^17,18^. In nanocrystalline materials, the theory of amorphous grain boundaries has persisted^19,20^. In recent studies, descriptions of grain boundaries in nanocrystalline materials have ranged from a highly disordered grain boundary structure described as frozen gas-like or amorphous, to the crystalline description now adopted for microcrystalline materials^21–27^. This discrepancy is thought to arise, in part, due to the different synthesis techniques and annealing histories used in the various experimental studies^28^; an “unrelaxed” nanocrystalline microstructure contains a significantly higher amount of atomic-level structural disorder in the grain boundary than is observed after aging^25^.

The structural disorder retained in a nanocrystalline material is present in the form of disordered regions, steps, ledges, extrinsic defects, and more complex dislocation content that arises due to excess defect concentrations and extensive GB/defect interactions during processing. In other words, grain boundaries can take on an ordered or disordered structure, depending on the structural units that make up these interfaces^28–32^. It has been shown through simulation that disordered, liquid-like grain boundaries can be formed^28,30,33^. In grain boundaries with misfits within 10 degrees deviation from the perfect twin, a stair structure can be found of structural units, which is a way to accommodate the deviation from a perfect twin configuration^34–37^. Materials produced by SPD techniques such as mechanical alloying^38^, high energy ball milling^38^, equal channel annual pressing^39,40^, high pressure torsion^40^, and accumulative roll-bonding^41^ are particularly susceptible to the formation of these distorted grain boundaries. Such grain boundaries have been termed ‘non-equilibrium’ partly due to their relative high energy, excess free volume, dislocation/disclination content, disordered atomic structure, and energetically metastable state as compared to their equilibrium counterparts.

A good measure of the deviation of a grain boundary from its equilibrium state is the additional degree of free volume as compared to that present in the equilibrium GB structure^28,30,31^. Excess free volume generally correlates with higher interfacial energy and atomic misfit, and is therefore a key physical attribute directly affecting many important GB properties, such as sliding, migration, and dislocation mediation processes^28,31,42–45^. Experimentally, the degree to which a grain boundary deviates from equilibrium is known to be dependent on the annealing treatment the sample undergoes. In a study of the role of grain boundary equilibrium on mechanical behavior, Swygenhoven *et al*.^28–30,46–49^ showed that annealing resulted in a more ordered grain boundary structure, and discovered that upon deformation, equilibrium grain boundaries exhibited less instantaneous plasticity than their non-equilibrium counterparts, and Tucker *et al*. showed using atomistic simulations that the resolved stress required to initiate grain boundary sliding, migration, and dislocation nucleation decreased with excess free volume.

In the current work, physical vapor deposition was used to prepare nanocrystalline iron films where the majority of grain boundaries were not at equilibrium, as identified in the TEM by a strong spreading of thickness extinction contours from the boundary^40^. The radiation tolerance of iron with non-equilibrium grain boundaries was directly compared to annealed iron films with grain boundaries significantly closer to equilibrium. Using *ex situ* ion irradiation, this work sought to directly compare the grain boundary sink strength, as defined by the ability of the grain boundary to influence the formation of defect clusters (and dislocation loops) within the grain interior. The relative sink strength of equilibrium and non-equilibrium grain boundaries was explored by comparing the behavior of annealed and as-deposited nanocrystalline iron films created by magnetron sputtering under *ex situ* ion irradiation. Nanocrystalline iron thin films were used for this study since (1) they provide a means to create specimens with either equilibrium or non-equilibrium grain boundaries, and (2) they permit direct characterization of radiation damage using transmission electron microscopy. The results of this evaluation are presented with a comparison to atomistic simulations of various model boundaries that were performed in tandem to understand the effect of free volume and grain boundary equilibrium on damage accommodation/annihilation.

## Methods

### Experimental Details

The iron films were deposited using magnetron sputtering of high purity iron onto sodium chloride substrates at Los Alamos National Laboratory^50^. Physical vapor deposition, like many bottom-up production methods for nanocrystalline materials, can result in the formation of non-equilibrium grain boundaries, especially when performed at low temperatures. The Structure Zone Model (SZM) proposed by Thornton^51^ describes the microstructure of an as-deposited sputtered film as a function of the temperature and argon pressure (both contribute to adatom mobility). According to the SZM, there is a critical temperature range that marks the transition zone, deemed *Zone T*, between the low temperature (*T/T*_*m*_ < 0.3) Z*one 1* microstructure with columnar structures defined by voided growth boundaries and the Z*one 2* structure (0.3 < *T/T*_*m*_ < 0.5) with columnar grains defined by metallurgical grain boundaries^51^. In the transition zone, the crystallites make physical contact, but the atoms do not have enough mobility to create the lowest possible energy configuration (i.e., an equilibrium grain boundary). A substrate temperature of 370 °C (0.36T_m_ of Fe) was chosen for deposition to target the Z*one T* in the Structure Zone Model proposed by Thornton^52^, thereby creating a specimen with non-equilibrium grain boundaries in the as-deposited condition. Each film was removed from the salt substrate and affixed to a 200 mesh nickel grid to create a free standing film specimen for transmission electron microscopy (TEM) experiments.

**Figure 1:**
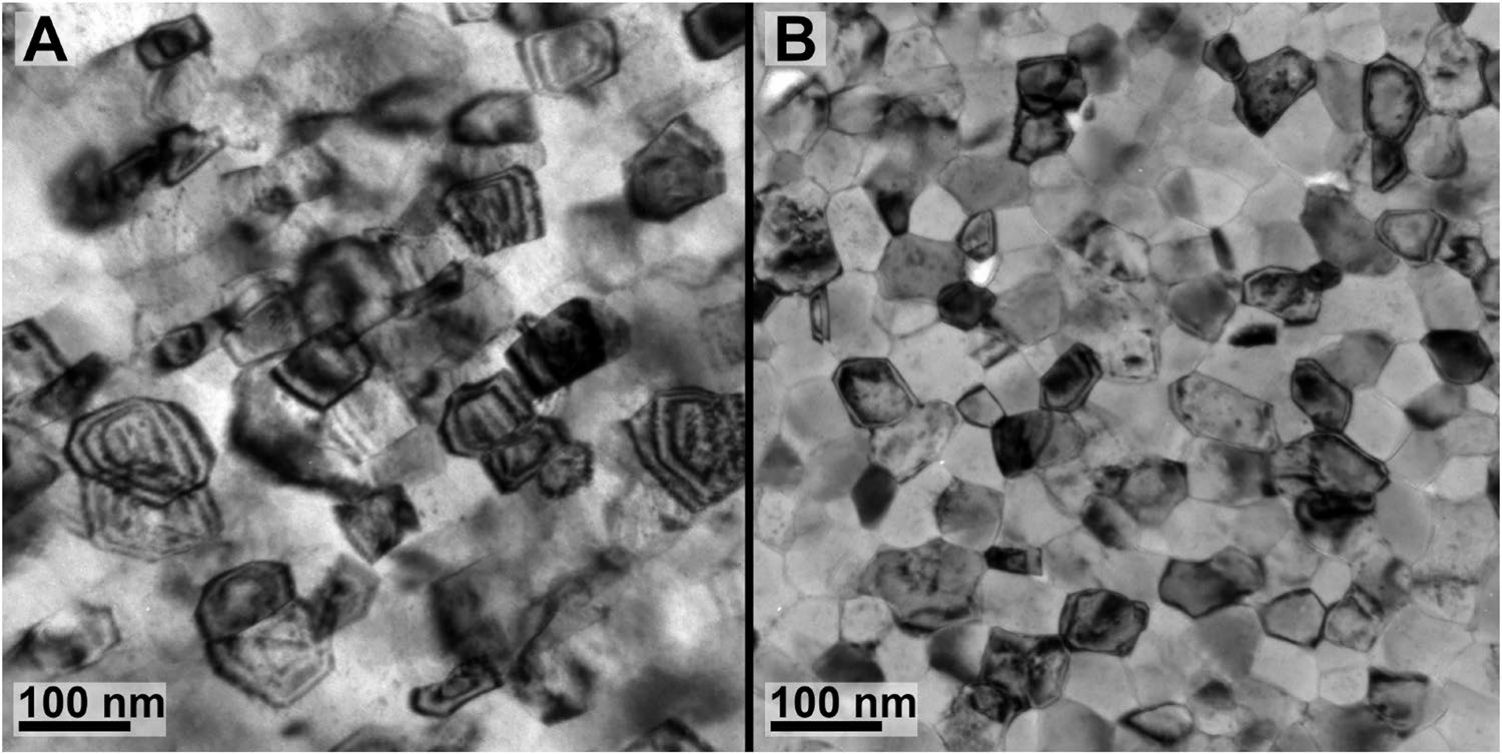
(**A**) The irradiation of an as-deposited nanocrystalline iron thin film to 5 dpa at 300 °C. Note the strong extinction bands, a classic sign of high amounts of strain at a non-equilibrium GB. (**B**) Irradiation of an annealed film to 5 dpa at 300 °C showing larger dislocation loops.

Non-equilibrium grain boundaries were identified in the nanocrystalline films using transmission electron microscopy (Fig. 1A), as the high level of elastic stresses and crystal lattice distortions near grain boundaries resulted in the spreading of thickness extinction contours^40^. To acquire equilibrium grain boundaries, the as-deposited films were then annealed to 600 °C for a period of approximately 5 minutes; this temperature was low enough to allow for recovery in the material without any measurable grain growth. After a 5 minute anneal at 625 °C, the extinction bands were found to be largely absent (Fig. 1B), indicating that the grain boundaries were driven toward equilibrium configurations.

**Figure 2:**
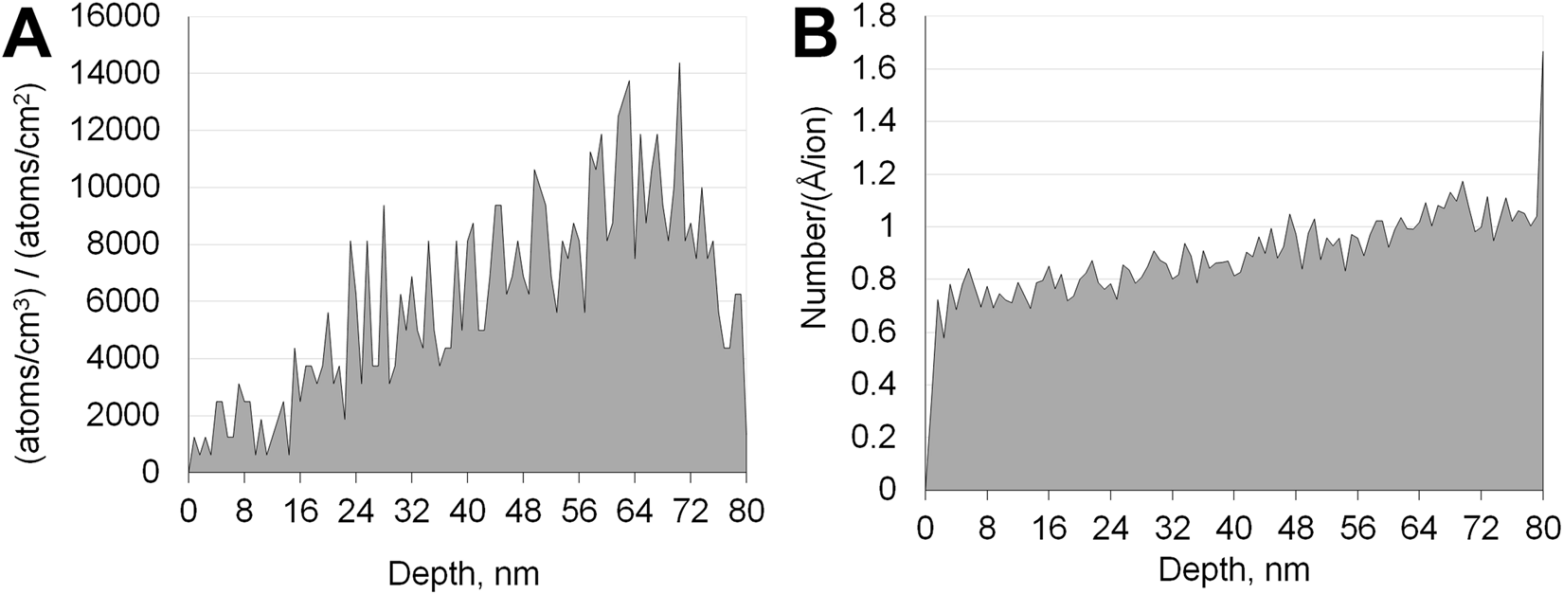
(**A**) The distribution of implanted Ar ions predicted by SRIM calculations. (**B**) The distribution of vacancies formed by the Ar ions as calculated by SRIM.

Samples were irradiated *ex situ* at 300 °C using 400 keV Ar ions at Los Alamos National Laboratory’s Ion Beam Materials Laboratory facility. The iron films used for irradiations were approximately 80 nm in thickness. The results of TRIM^53^ calculations for 400 keV Ar ions predicted a 94.9% transmission rate. As calculated by SRIM, the projected range of 400 MeV Ar ions in iron, coincident with the peak damage and implantation, would be 177 nm or more than double the thickness of the films used in this study. Figure 2A shows the distribution of argon atoms implanted into the film during irradiation. The damage resulting from irradiation with 400 keV Ar ions was predicted to be nearly uniform through-thickness, as shown in Fig. 2B.

Each sample was stabilized at 300 °C before being irradiated with 400 keV Ar^2+^ ions. As-deposited (non-annealed) and annealed nanocrystalline iron films were irradiated *ex situ* to a dose of 4 × 10^15^ ions cm^−2^ (nearly 5 dpa as calculated by TRIM^53^) using 400 keV Ar ions. Post-irradiation imaging was performed using a JEOL LaB_6_ TEM at a 200 kV accelerating voltage, which is below the knock-on damage threshold for iron. Defect densities were determined using an areal density in many grains that shared a common diffracting condition. Size measurements were performed by measuring the length of the strain field for each visible defect cluster present.

### Atomistic Simulations

Molecular Dynamics (MD) simulations were performed in order to better understand the processes occurring on timescales too short to capture using *in situ* TEM. For the simulations, the iron potential created by Mendelev *et al*.^54^ was used. Primary knock-on atoms (PKAs) with energy of 3 keV were created at 3 nm from the grain boundary plane in a bicrystalline simulation. The initial PKA velocity was oriented normal to and towards the boundary plane. This initial energy and distance were chosen to create a small, localized cascades within the grain boundaries. The simulation was run at 300 K, using the isothermal-isobaric (NPT) thermostat with 0 pressure, to observe the evolution of the cascade at two different grain boundaries: a Σ5 (210) < 001 > symmetric tilt grain boundary (~53° misorientation angle), and another grain boundary with the same 5 degrees of freedom but with artificially introduced excess free volume to create a non-equilibrium instantiation of the Σ5 (210) < 001 > symmetric tilt grain boundary^10,30^. One thousand PKAs were simulated in parallel for each bicrystalline simulation cell in order to investigate the stochastic nature of damage accommodation in a statistical sense of the simulated grain boundaries.

## Results and Discussion

As-deposited iron films contained a columnar grain structure with an average grain size between 30 and 50 nm, depending on the particular TEM specimen measured. Annealing the films had no observable effect on the grain size. Figure 1 shows examples of iron films irradiated in both the as-deposited (Fig. 1A) and annealed (Fig. 1B) condition. Grain boundaries in the as-deposited films, before and after irradiation, exhibited strong extinction bands - a classic sign of high amounts of strain at a non-equilibrium GB. Given that the 370 °C substrate temperature utilized for the deposition is 0.36T_m_ of Fe, this type of morphology is consistent with the classic *Zone T* in the Structure Zone Model proposed by Thornton^51,52^. Short anneals of the iron films prior to irradiation resulted in a significant decrease in the presence of extinction bands at the grain boundary (visible in the irradiated film in Fig. 1B), indicating that enough thermal energy was available to relax the grain boundaries closer to an equilibrium configuration.

**Figure 3:**
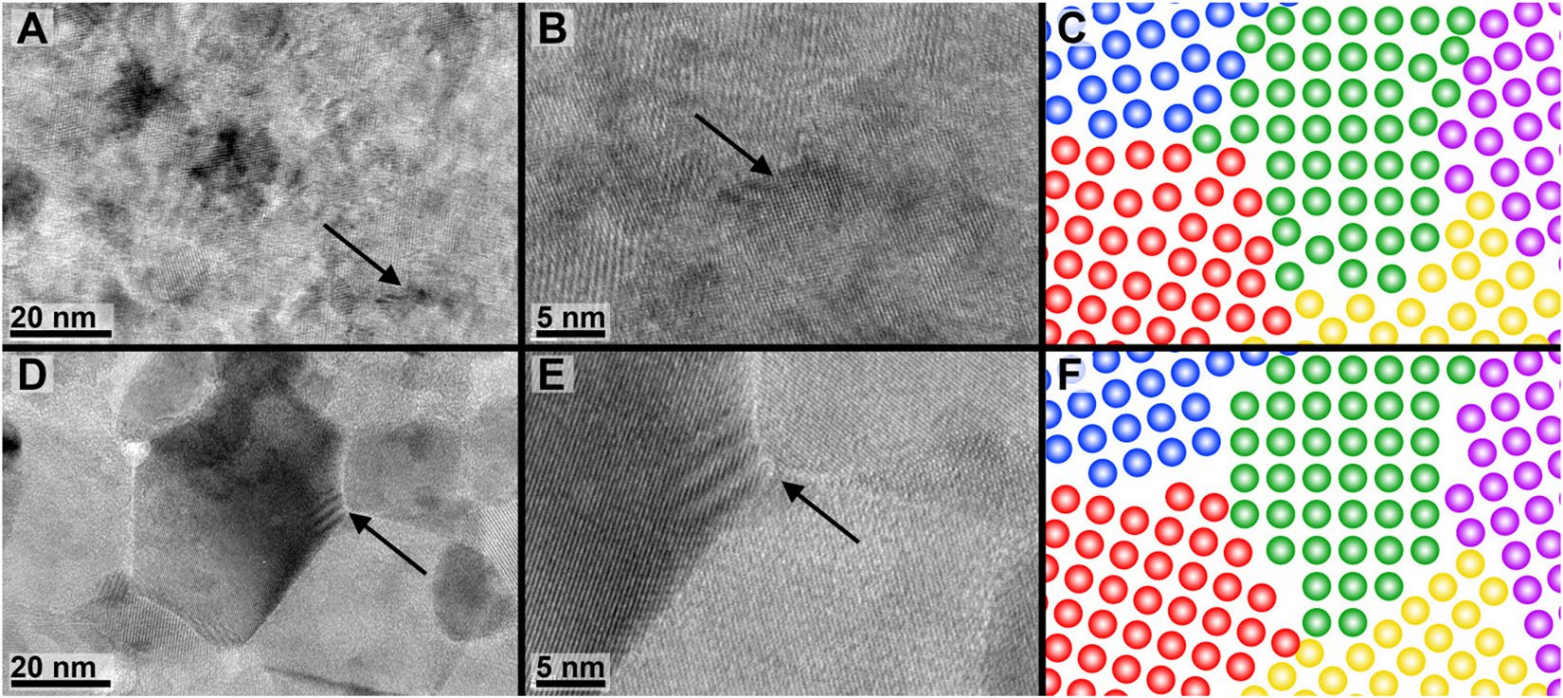
The black arrow in (**A**) indicates the location of the HRTEM image in (**B**) which shows that the as-deposited iron film contains poorly defined grains with ill-defined grain boundaries. These grain boundaries contain atoms in non-equilibrium positions (schematically depicted in **C**), resulting in excess free volume and dislocation content. (**D**) Shows the same film annealed to 600 °C for 10 minutes generating a well-defined grain structure. The black arrow in (**D**) indicates the location of the HRTEM image in (**E**) where near-equilibrium grain boundary structure is visible in the higher magnification image of the triple junction. Annealing allowed the atoms at the grain boundaries to assume equilibrium positions and reduce the excess free volume, resulting in grain boundaries that consist largely of ordered dislocations. (**F**) Schematic representation of the equilibrium grain boundary structure.

High resolution transmission electron microscopy (HR-TEM) imaging of nanocrystalline iron films (Fig. 3) confirmed that the as-deposited films contained ill-defined grain boundaries. An example of such a grain boundary is indicated by the black arrow in Fig. 3B. The black arrow in Fig. 3A shows where this grain boundary is in a lower magnification view. The annealed film specimens, which show substantially decreased extinction band widths at low magnification, also show atomically well-defined grain boundaries with narrow interfaces (less than 1 nm) that signify near-equilibrium structure (Fig. 3D and E) in HR-TEM.

**Figure 4:**
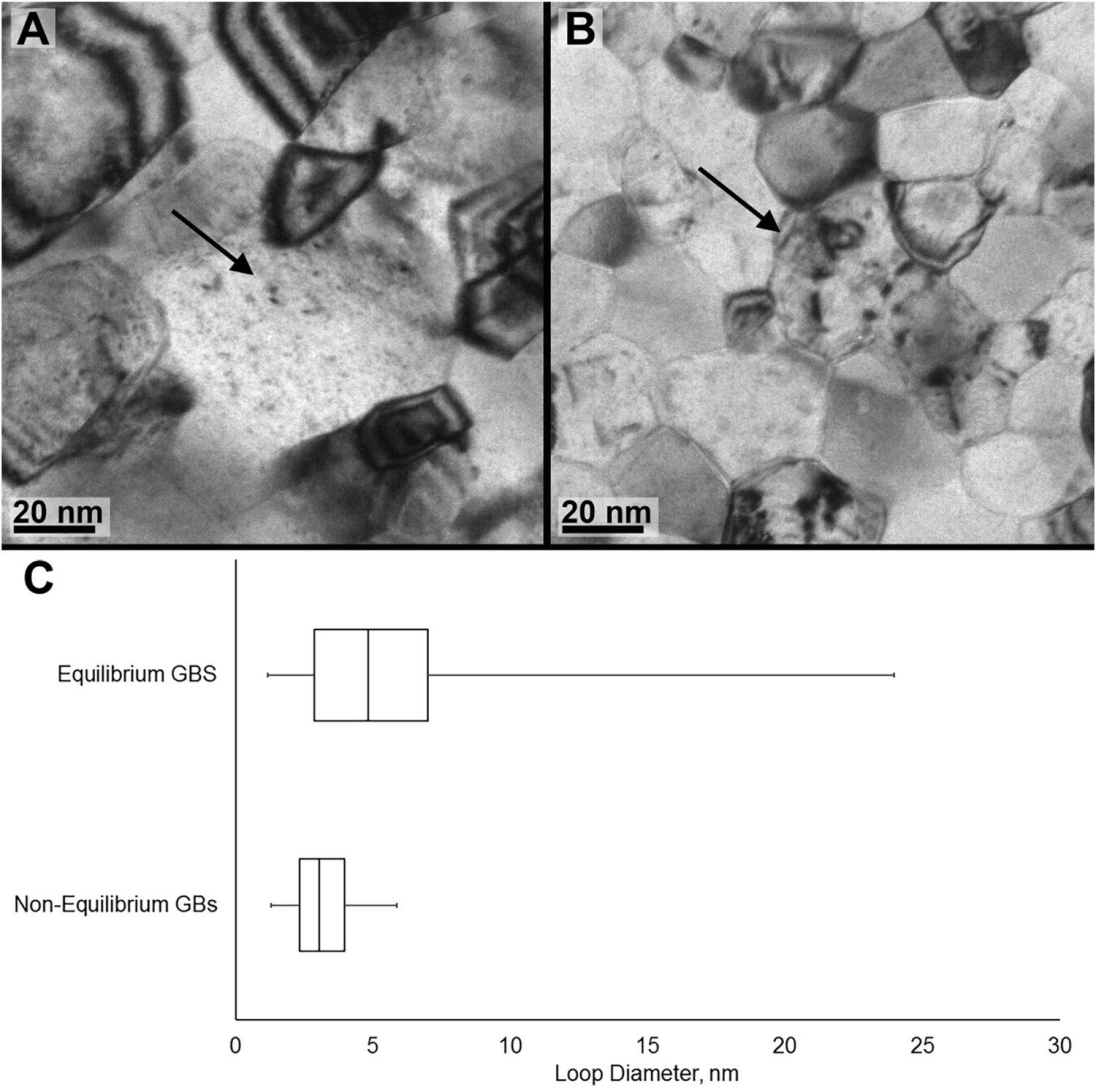
A comparison of the size and density of dislocation loops in (**A**) non-annealed and (**B**) annealed nanocrystalline iron after *ex situ* irradiation to 4 × 10^15^ ions cm^−2^ using 400 keV Ar ions. Quantitative measurements of loop diameter are shown as a box and whisker plot (**C**) for the each of the grains indicated with black arrows in A and B.

After irradiation to 5 dpa, marked differences were seen in the irradiation response of the equilibrium versus the non-equilibrium grain boundaries (Fig. 4). The nanocrystalline iron containing primarily non-equilibrium boundaries demonstrates a greater propensity for point defect absorption as compared to the annealed iron with equilibrium grain boundary structure. This is present as limited dislocation loop growth observed in the non-annealed films. While the microstructure of both materials contained primarily small dislocation loops or clusters, which may be expected based on prior results in nanocrystalline materials^5–7^, the defect densities and dislocation loop sizes differed significantly. Quantitative defect analysis results show that the defect cluster density in as-deposited (non-equilibrium) nanocrystalline iron films is approximately 4000 defect clusters/µm^2^, while annealed nanocrystalline iron contains approximately 7000 defect clusters/µm^2^.

In addition to a larger population of defect clusters, annealed films with equilibrium grain boundaries also contained larger dislocation loops (Fig. 4C). The average dislocation loop size in both films is similar; in the equilibrium grain boundary films the average is about 6 nm, compared to the average loop size of about 4 nm in the films with non-equilibrium grain boundaries. The distinction between the two films is the range of dislocation loops present. In films with non-equilibrium grain boundaries, dislocation loop diameters of up to about 20 nm were common (Fig. 4B). On the other hand, in the as-deposited films with non-equilibrium grain boundaries, defect clusters are predominately 2–6 nm in diameter, and no larger (>6 nm) loops were found (Fig. 4A). The larger loop population and greater loop size in the annealed iron with equilibrium grain boundaries indicates that a higher fraction of point defects survived to create larger dislocation structures. This is consistent with the fact that nucleation and growth of defect clusters depends upon the survival rate of the defects from the cascade (displacement efficiency), the form and mobility of the interstitials and vacancies created (production bias^10,55,56^), and the interstitial-vacancy recombination rate. Displacement efficiency can be strongly affected by the presence of grain boundaries. Cascade events that occur at or near a grain boundary lose a large fraction of the interstitials formed during the cascade due to spontaneous recombination with the boundary^48,57–60^. Additional interstitials and vacancies can be removed via non-diffusive^48,58,61^ and diffusive^62,63^ mechanisms, starving the nucleated clusters of the additional point defects required for growth (into, for example, dislocation loops). Work by Samaras *et al*.^48,58^ showed that the interstitials are attracted to regions of high tensile stress and accommodated by the free volume, indicating that both of these features are required in the boundary for it to act as an efficient sink.

**Figure 5:**
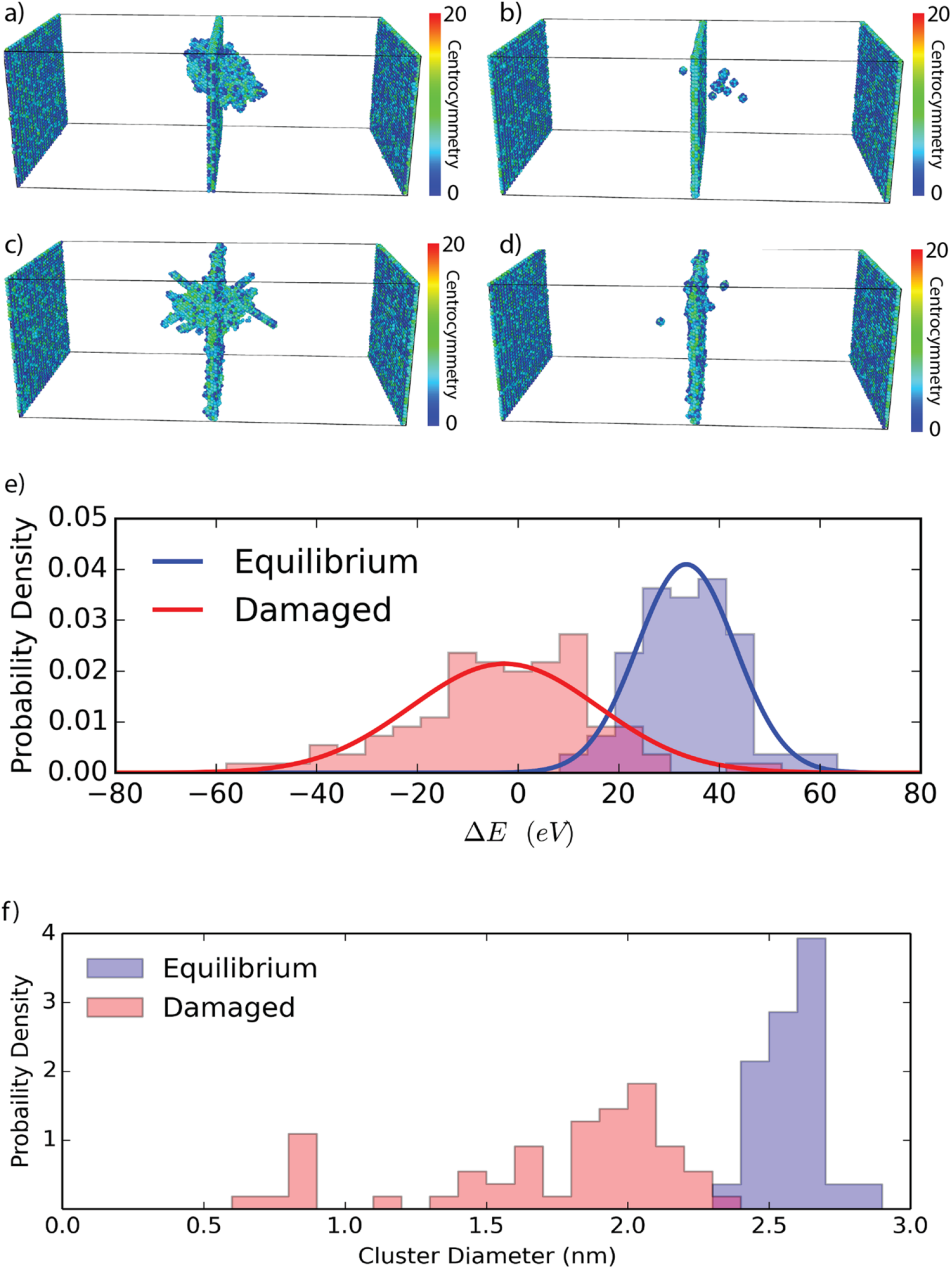
Cascade collapse into a Σ5 (210) < 001 > symmetric tilt grain boundary at room temperature. Atoms colored according to potential energy. (**a**) Peak damage after the cascade for the equilibrium GB structure. (**b**) Remaining defects after 30 ps thermal equilibration. (**c**) Peak damage in an iron bicrystal with a non-equilibrium Σ5 (210) < 001 > symmetric tilt GB (NEGB). (**d**) Remaining defects after 30 ps thermal equilibration. (**e**) Distributions of excess energy of 1000 PKAs after 30 ps relaxation. (**f**) Distribution of defect cluster diameters after 1000 events.

Molecular dynamics simulations reveal excess free volume in non-equilibrium boundaries does contribute to a decreased survival rate of point defects from nearby simulated cascades. For a 3 keV primary knock-on atom (PKA) created at 3 nm from an equilibrium and non-equilibrium Σ5 (210) <001> symmetric tilt grain boundary (53**°** misorientation angle), a small region of locally amorphous atoms quickly recrystallizes but leaves many point defects behind. These point defects spontaneously recombine both with equilibrium and non-equilibrium boundaries, but at varying rates. Figure 5 shows typical radiation cascades in both boundaries (Fig. 5a,c) and the distribution of point defects after 30 ps equilibration (Fig. 5b,d), where in both cases BCC-coordinated atoms, as identified by common neighbor analysis, are rendered invisible. Many more point defects remain in lattice regions adjacent to equilibrium boundaries as compared to non-equilibrium.

The aggregate effect of one thousand PKAs is presented in Fig. 5e where the equilibrium boundary always experiences a net gain in energy due to defect absorption by the boundary, suggesting the presence of both point defects in the crystalline region and a higher energy GB structure after the cascade. In contrast, the non-equilibrium “damaged” GB showed no proclivity for either energy gain or loss, but on average demonstrated no net change in energy. Figure 5f similarly shows that remaining defect cluster sizes are significantly larger in equilibrium boundaries as well. The increased energy gain of equilibrium boundaries can be explained both by the increased concentration of remaining defects, and also by structural changes within the boundaries. While equilibrium GBs must, by definition, gain energy while undergoing structural changes undergoing zero pressure/stress, NEGBs can either gain or lose energy. As a consequence, when a boundary is altered by the absorption of point defects, the degree and character or structural changes is dependent on boundary character, (i.e. equilibrium vs. non-equilibrium). Furthermore, as shown in recent work by Foley and Tucker^10^, under continuous damage accumulation, an equilibrium grain boundary will evolve through many non-equilibrium states until a steady “damaged” state is found that is higher in both energy and free volume than the equilibrium state. Here, the distribution of final energies is also significantly broader for non-equilibrium boundaries, suggesting that a cascade in a non-equilibrium boundary samples a wider range of boundary states than in an equilibrium boundary. The absorption of point defects can both lower and raise the energy, with no net change on average, of a non-equilibrium boundary indicates that, in addition to a higher sink-efficiency, non-equilibrium systems can provide a route towards the engineering of systems with steady-state properties while providing high radiation tolerance.

In summary, experiments testing the ability of equilibrium and non-equilibrium grain boundaries to act as defect sinks under irradiation were carried out using nanocrystalline iron films and compared to molecular dynamics simulations in iron. Results of TEM experiments indicated that more defects were being absorbed by the non-equilibrium grain boundaries, resulting in a lower population of smaller dislocation loops. The size of the dislocation loops being much smaller for the structure with non-equilibrium grain boundaries suggests that, due to their higher energy and free volume, act as stronger defect sinks. The molecular dynamics simulations support the assentation that non-equilibrium grain boundaries act as more efficient sinks for defects and undergo less dramatic changes in structure because of irradiation, suggesting microstructures with higher fractions of such boundaries demonstrating efficient damage accommodation could be utilized for future radiation tolerant materials.
